# Disentangling the Multidimensional Structure of Self-Rated Health: The Predominant Role of Physical Capability in Older Japanese Adults

**DOI:** 10.7759/cureus.97107

**Published:** 2025-11-17

**Authors:** Hirotomo Shibahashi, Kanta Ohno, Yosuke Seike, Shinpei Ikeda

**Affiliations:** 1 Occupational Therapy, Department of Rehabilitation, School of Health Sciences, Tokyo University of Technology, Tokyo, JPN; 2 Faculty of Social Welfare, University of Kochi, Kochi, JPN; 3 Occupational Therapy, Department of Rehabilitation, Faculty of Medical Sciences, Shonan University of Medical Sciences, Kanagawa, JPN

**Keywords:** japanese older adults, physical capability, principal component analysis, psychosocial factors, self-rated health

## Abstract

Background

Self-rated health (SRH) is a widely used global health indicator and a strong predictor of morbidity, disability, and mortality in older adults. Unlike objective clinical measures, SRH represents a holistic appraisal of physical, psychological, and social aspects of health. Although prior studies have identified sociodemographic characteristics and multimorbidity as key determinants of poor SRH, the contribution of broader psychosocial and functional dimensions remains unclear. As these domains are typically measured using multiple interrelated indicators, their independent effects are often obscured by redundancy and multicollinearity. To clarify the underlying structure of SRH and disentangle the relative influence of health-related dimensions, this study aimed to empirically derive core components from a wide range of health and lifestyle variables and examine their associations with SRH in community-dwelling older adults in Japan.

Methods

This population-based cross-sectional study used secondary data from the 2017 Survey on Health and Life of Older Adults in Ayase City, Japan, including 1,821 community-dwelling adults aged 65 years or older. Fifty-eight items covering physical, psychological, and social domains were analyzed, incorporating standardized measures such as the Geriatric Depression Scale, Tokyo Metropolitan Institute of Gerontology Index of Competence, and the Motor Fitness Scale. Principal component analysis (PCA) was applied to identify latent components, and multiple imputation addressed missing data. Multivariable logistic regression examined associations between component scores and SRH, adjusting for age, sex, and multimorbidity. All analyses were conducted using R.

Results

Among the 1,821 older adults analyzed, 73.4% reported high and 26.6% reported low SRH. Participants with low SRH were older, had lower educational attainment, and were more likely to have three or more physician-diagnosed conditions (χ² = 218.87, P < 0.001). PCA identified four components explaining 49.9% of the variance: physical capability, social well-being, mobility patterns, and social participation. In multivariable logistic regression adjusted for age, sex, and number of physician-diagnosed conditions, higher physical capability was strongly associated with better SRH (odds ratio (OR) 2.63, 95% confidence interval (CI) 2.17-3.19, P < 0.001), whereas mobility patterns showed a modest negative association (OR 0.85, 95% CI 0.74-0.98, P = 0.024). Social well-being and social participation were not significantly associated with SRH.

Conclusion

Physical capability was the most influential determinant of SRH among community-dwelling older adults, whereas greater reliance on public transportation was modestly associated with poorer perceived health. Social well-being and social participation did not show independent associations after accounting for physical function and the number of physician-diagnosed conditions. These findings highlight the central importance of physical function and mobility independence in shaping health perception. Strategies that promote motor fitness, support independent transportation, and manage multiple conditions may enhance SRH and overall well-being in aging populations.

## Introduction

Self-rated health (SRH) is widely recognized as a robust predictor of morbidity, disability, and mortality among older adults and is increasingly regarded as a global health indicator [1-3]. Unlike objective clinical measures, SRH reflects a holistic appraisal of physical, psychological, and social domains of health. Because of its simplicity and predictive validity, SRH has been incorporated into large-scale population surveys and has informed public health policies worldwide [4-6]. A recent systematic review reported that SRH integrates medical, functional, mental, and social dimensions, capturing how individuals perceive and synthesize multiple facets of their well-being into a single, global judgment [1]. In Japan, national health promotion initiatives, such as Health Japan 21, emphasize not only the maintenance of physical health but also active participation in community activities and the strengthening of social connectedness as integral components of healthy aging [7].

Previous studies have shown that sociodemographic characteristics, including female sex, older age, and lower educational attainment, together with medical conditions, particularly multimorbidity, are strongly associated with poor SRH [8-10]. Beyond these conventional determinants, psychosocial and functional domains, including social participation, community connectedness, and physical capability, have also been implicated [11,12]. However, these domains are typically assessed using multiple interrelated indicators, which often exhibit multicollinearity and redundancy, complicating interpretation.

Although prior research highlights the multifaceted nature of SRH [13], the relative contribution of different health-related dimensions remains unclear when traditional covariates, such as age and multimorbidity, are considered. In particular, few studies have empirically reduced complex sets of physical, psychological, and social indicators to core components [14]. Whereas the previous analysis used a decision tree model to explore hierarchical and nonlinear determinants of SRH [15], the present study adopted a complementary, data-reduction approach to identify latent dimensions underlying multiple health-related indicators.

This methodological progression from exploratory to integrative analysis was intended to provide a more parsimonious understanding of the multidimensional nature of SRH. This gap hampers the understanding of whether SRH primarily reflects objective morbidity or broader psychosocial resources.

To address this gap, the present study aimed to identify the principal components underlying health-related variables among community-dwelling older adults in Japan and examine their independent associations with SRH. By applying principal component analysis (PCA) in combination with multivariable regression models, we sought to clarify whether these empirically derived components explain variation in SRH beyond the effects of age, sex, and number of physician-diagnosed conditions.

Our previous study using the same dataset identified hierarchical determinants of SRH through decision tree analysis, highlighting the predominant roles of physical capability, depressive symptoms, and daily competence [15].

Building upon those findings, the present study aimed to disentangle the multidimensional structure underlying SRH by applying PCA and logistic regression to clarify the latent health domains influencing perceived health.

## Materials and methods

Study participants

This population-based, cross-sectional study utilized secondary data from the Survey on Health and Life of Older Adults, which had been conducted by Ayase City, Kanagawa Prefecture, Japan, as part of its public health activities. The self-administered questionnaire was distributed by mail between June 28 and July 9, 2017. Of the 3,058 individuals invited by the municipality, 1,899 returned completed questionnaires (response rate, 62.1%). The authors performed a secondary analysis using anonymized data provided by Ayase City.

Inclusion criteria were community-dwelling adults aged ≥65 years who completed and returned the survey. Exclusion criteria included respondents with missing data on SRH or with substantial nonresponse across survey items. After these exclusions, 1,821 participants were included in the final analytic sample.

Because the sampling frame comprised only older adults, all analyses were conducted within this age-defined population to examine associations between sociodemographic and health-related variables and SRH.

As this study analyzed secondary data from an existing municipal survey, a priori sample size calculation was not performed. Ethical approval was obtained from the Ethics Committee of J. F. Oberlin University (approval number: 17007). Consistent with the committee’s policy, the return of a completed questionnaire was considered the provision of informed consent.

Measurements

SRH was assessed using a single question: “How would you describe your current health status?” Respondents selected one of four response options: very healthy, fairly healthy, not very healthy, or not healthy. For the purpose of analysis, responses of very healthy and fairly healthy were classified as high SRH, whereas not very healthy and not healthy were classified as low SRH. This single-item measure corresponds to the widely used format for assessing SRH in population-based surveys and has been validated as a reliable indicator of subjective health perception among older adults in Japan.

For the present analysis, 58 items were selected to comprehensively represent the physical, psychological, and social dimensions that have been reported to be associated with SRH [16]. These items served as the study parameters for subsequent analyses. The selection focused on variables theoretically and empirically linked to functional ability, psychological well-being, and social engagement, while excluding administrative or context-specific items unrelated to individual health perception.

A total of 58 items covering multiple domains of health, functional ability, and social participation were analyzed. The standardized instruments included the five-item Geriatric Depression Scale (GDS-5; score range, 0-5) [17], the five-item Tokyo Metropolitan Institute of Gerontology Index of Competence (TMIG-IC; score range, 0-5) [18], and the 14-item Motor Fitness Scale (MFS; score range, 0-14) [19]. The Japanese version of the GDS-5 used in this study was adapted from the short form originally developed and validated by Hoyl et al. [17], which includes items assessing life satisfaction, boredom, preference for staying at home, sense of purposelessness, and helplessness. The TMIG-IC was administered as a five-item short form adapted from the original 13-item version developed by Koyano et al. [18]. In addition, 34 municipality-specific items developed by Ayase City for health and welfare planning were included to assess social and lifestyle domains, such as social network size, neighborhood trust and cohesion, frequency of community participation, frequency of going outdoors and exercising, and primary modes of transportation (walking, bicycle, public transportation, private car, and mobility aids). These items have been used in previous community surveys conducted by the municipality. A complete list of all 58 items is presented in the Appendix.

The TMIG-IC and the MFS are widely used standardized instruments for evaluating competence and physical fitness among older adults in Japan. Both instruments are publicly available for non-commercial academic use, and formal permission was not required.

Statistical analysis

PCA was applied to the 58 items to extract latent components representing the multidimensional nature of health. Missing data were addressed using multiple imputation by chained equations, generating 20 imputed datasets with 50 iterations each [20]. The component structure was derived from the first imputed dataset using a polychoric correlation matrix and replicated across 20 imputed datasets to compute component scores [21,22]. These scores were merged with age, sex, and the number of physician-diagnosed conditions, and multivariable logistic regression models with SRH as the dependent variable were fitted to each dataset. Regression estimates were pooled across imputations using Rubin’s rules, as implemented in the mitools package (MIcombine function). All analyses were conducted using R (version 4.5.1; R Foundation for Statistical Computing, Vienna, Austria).

## Results

Of the 1,821 participants analyzed, 1,337 (73.4%) reported high SRH (responding “very healthy” or “fairly healthy”), and 484 (26.6%) reported low SRH (“not very healthy” or “not healthy”) (Table 1).

**Table 1:** Participant characteristics grouped by SRH Values are presented as numbers (percentages) unless otherwise indicated. Age is expressed in years (median, interquartile range (IQR_). SRH: self-rated health; IQR: interquartile range; †: Mann-Whitney U test (U-value shown); ‡: Chi-square test (χ²-value shown)

Variable	High-SRH group (n = 1337)	Low-SRH group (n = 484)	Test statistic	P-value
Age (years, median, IQR)	74 (70-78)	77 (72-81)	U = 253247	<0.001†
Sex (%)				
female	726 (54.3)	260 (53.7)	χ2 = 0.027831	0.832‡
male	611 (45.7)	224 (46.3)		
Years of education (%)				
6 years or less	6 (0.4)	12 (2.5)	χ2 = 45.905	<0.001‡
7–9 years	211 (15.8)	121 (25.0)		
10–12 years	622 (46.5)	213 (44.0)		
13 years or more	457 (34.2)	116 (24.0)		
No response	41 (3.1)	22 (4.5)		
Number of diagnosed conditions (%)				
0-2	1172 (87.7)	272 (56.2)	χ2 = 218.87	<0.001‡
3-5	159 (11.9)	194 (40.1)		
6-8	6 (0.4)	18 (3.7)		

Participants with low perception were significantly older than those with high perception (median 77 years, interquartile range (IQR) 72-81 vs. 74 years, IQR 70-78; Mann-Whitney U = 253,247, P < 0.001). They also had lower educational attainment (χ² = 45.91, P < 0.001). The distribution of physician-diagnosed conditions differed markedly between groups (χ² = 218.87, P < 0.001). Among participants with low SRH, 272 (56.2%) had two or fewer conditions, 194 (40.1%) had three to five, and 18 (3.7%) had six to eight, compared with 1,172 (87.7%), 159 (11.9%), and six (0.4%), respectively, among those with high SRH. No significant difference was observed in sex distribution (P = 0.832). Details of physician-diagnosed conditions are provided in Table 2.

**Table 2:** Distribution of physician-diagnosed conditions among participants, stratified by SRH perception group (multiple responses allowed) Values are presented as number of participants (n). Because multiple responses were permitted, the numbers in each category represent absolute counts rather than mutually exclusive proportions. Therefore, these figures should be interpreted as descriptive distributions rather than comparative statistics. No statistical test was applied. SRH: self-rated health; COPD: chronic obstructive pulmonary disease; BPH: benign prostatic hyperplasia

Variable	High SRH perception group (n = 1337)	Low SRH perception group (n = 484)
Hypertension	503	217
Stroke (including cerebral hemorrhage, cerebral infarction)	23	46
Osteoporosis	63	76
Rheumatoid arthritis	19	23
Spinal canal stenosis	47	54
Osteoarthritis	84	63
Fracture	9	17
Cataract	102	87
Glaucoma	65	37
Hearing loss	64	55
Diabetes mellitus	137	96
Hyperlipidemia	154	72
Angina pectoris	31	36
Myocardial infarction	16	15
Bronchial asthma	35	17
Pneumonia	4	12
COPD	5	3
Renal failure	7	22
BPH	65	43
Gastric or duodenal ulcer	22	17
Cancer	27	66
Dementia	14	25
Parkinson's disease	6	11
Other	116	73

**Figure 1:**
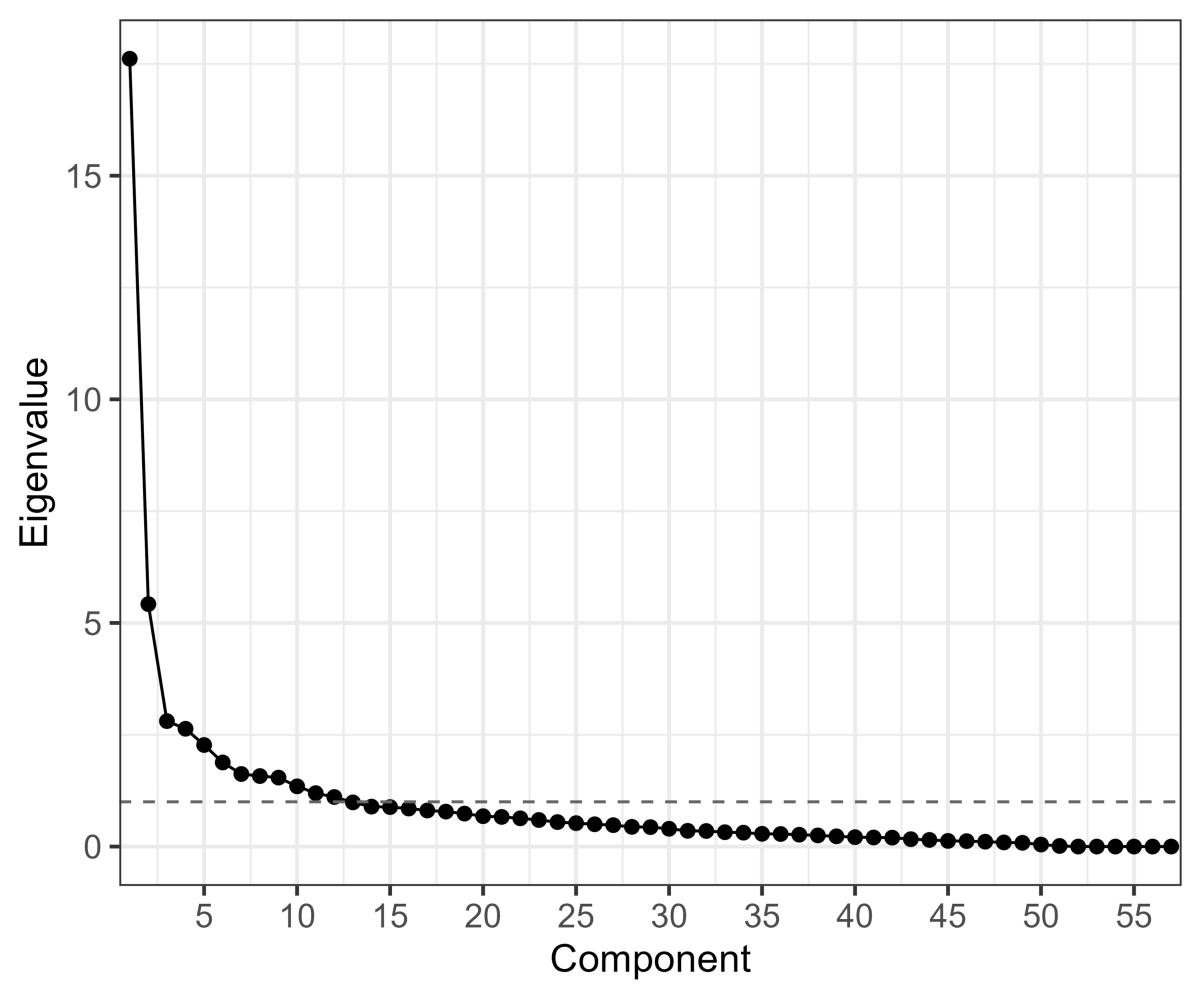
Scree plot of eigenvalues from the PCA The scree plot displays the eigenvalues corresponding to each principal component derived from the analysis of 58 health- and lifestyle-related variables. The point of inflection, indicated by a marked decline in eigenvalues, suggests that four components adequately capture the underlying structure of the dataset. PCA: principal component analysis

PCA identified four components with eigenvalues >1, explaining 49.9% of the variance (PC1 = 30.9%, PC2 = 9.5%, PC3 = 4.9%, and PC4 = 4.6%). The scree plot indicated an inflection after the fourth component, supporting retention of four components (Figure 1).

The component loadings (Table 3) indicated four interpretable dimensions: physical capability, representing motor fitness and IADL-related items such as jumping, running, and lifting; social well-being, characterized by fewer social contacts but higher life satisfaction, trust, and perceived mutual help; mobility patterns, reflecting greater reliance on public transportation as opposed to private or assistive modes; and social participation, encompassing involvement in community, hobby, and volunteer activities.

**Table 3:** Component loadings of 58 health- and lifestyle-related variables across four principal components Loadings represent correlation coefficients between each variable and the extracted component, derived from PCA. Full item list, including rarely endorsed transportation modes (e.g., use of motorized four-wheeled vehicles), is provided in Appendix. PCA: principal component analysis

Variable	Physical capability	Social well-being	Mobility patterns	Social participation
Feels bored every day	-0.37	-0.34	–	-0.31
Prefers staying at home to going out or trying new activities	-0.45	–	–	-0.39
Feels that life is not worthwhile	-0.43	-0.33	–	-0.32
Feels helpless	-0.5	-0.36	–	–
Able to climb stairs	0.84	–	–	–
Able to climb stairs without shortness of breath	0.61	–	–	–
Able to jump	0.87	–	–	–
Able to run	0.85	–	–	–
Able to overtake others while walking	0.81	–	–	–
Able to continue walking for more than 30 minutes	0.85	–	–	–
Able to carry a bucket filled with water	0.85	–	–	–
Able to lift a 10-kg bag of rice	0.82	–	–	–
Able to lift a fallen bicycle	0.86	–	–	–
Able to open the lid of a wide-mouthed jar (e.g., jam jar)	0.68	–	–	–
Able to touch the floor from a standing position without bending the knees	0.39	–	–	–
Able to put on socks, trousers, or a skirt while standing without support	0.78	–	–	–
Able to stand up from a chair without using hands	0.84	–	–	–
Able to stand on tiptoe without holding onto anything	0.82	–	–	–
Able to go out alone using public transportation	0.67	–	0.52	–
Able to manage bank deposits and withdrawals	0.5	–	0.41	–
Able to provide advice or counsel to family members or friends	0.45	0.44	–	–
Able to shop for daily necessities	0.71	–	0.35	–
Frequency of going outdoors	0.45	–	–	–
Frequency of exercise	0.33	–	–	–
Usual mode of transportation when going out: Bicycle	-0.37	–	–	–
Usual mode of transportation when going out: Car (as a passenger)	0.39	–	–	–
Usual mode of transportation when going out: Car (self-driving)	-0.58	–	0.32	–
Usual mode of transportation when going out: Rollator (wheeled walker)	0.61	–	–	–
Usual mode of transportation when going out: Wheelchair	0.72	–	0.47	–
Usual mode of transportation when going out: Other	0.51	–	–	–
Visits friends at their homes	0.31	0.42	–	0.36
Satisfaction with life	–	0.37	–	–
Number of relatives contacted at least once a month (by phone or in person)	–	-0.71	–	–
Number of relatives with whom one can comfortably discuss personal matters	–	-0.79	–	–
Number of relatives who can be relied upon for help in times of need	–	-0.74	–	–
Number of friends contacted at least once a month (by phone or in person)	–	-0.61	–	-0.31
Number of friends with whom one can comfortably discuss personal matters	–	-0.64	–	–
Number of friends who can be relied upon for help in times of need	–	-0.67	–	–
Frequency of conversations with neighbors	–	-0.49	–	-0.33
Attachment to the community	–	0.4	–	0.39
Many neighbors are of a similar generation or lifestyle	–	0.42	–	0.31
Frequently interacts with people of similar background in daily life	–	0.54	–	0.41
People in the community can be trusted	–	0.5	–	–
People in the community are willing to help each other	–	0.52	–	–
Usual mode of transportation when going out: Walking	-0.41	–	-0.61	–
Usual mode of transportation when going out: Motorcycle	–	–	0.33	–
Usual mode of transportation when going out: Bus	–	–	-0.77	–
Usual mode of transportation when going out: Train	-0.39	–	-0.67	–
Usual mode of transportation when going out: Taxi	0.43	–	-0.5	–
Frequently interacts with people of different backgrounds in daily life	–	–	–	0.32
Willingness to contribute to the local community	–	–	–	0.56
Frequency of participation in community events	–	–	–	0.66
Frequency of participation in neighborhood association activities	–	–	–	0.61
Frequency of participation in senior club activities	–	–	–	0.67
Frequency of participation in hobby groups	–	–	–	0.61
Frequency of activities involving sharing one’s skills or experiences with others	–	–	–	0.64
Frequency of participation in volunteer activities	–	–	–	0.73

These four components were subsequently entered into multivariable logistic regression models to examine their independent associations with SRH (Table 4).

**Table 4:** Multivariable logistic regression models for the association between principal component scores and SRH Physical capability, social well-being, mobility patterns, and social participation were derived from PCA. Results were obtained from multivariable logistic regression analyses adjusted for age, sex, and number of physician-diagnosed conditions. SRH: self-rated health; OR, odds ratio; CI, confidence interval; PCA: principal component analysis

Variable	OR	95%CI	P value
Physical capability	2.63	2.17-3.19	<0.001
Social well-being	0.96	0.83-1.11	0.558
Mobility patterns	0.85	0.74-0.98	0.024
Social participation	1.01	0.88-1.16	0.849
Age (years)	0.97	0.95-0.99	0.019
Gender (male vs. female)	1.28	0.97-1.71	0.085
Number of physician-diagnosed conditions	1.78	1.58-2.00	<0.001

In multivariable logistic regression analyses adjusted for age, sex, and number of physician-diagnosed conditions (Table 4), higher physical capability scores were strongly associated with better SRH (OR 2.63, 95% CI 2.17-3.19, P < 0.001). Mobility patterns showed a modest negative association (OR 0.85, 95% CI 0.74-0.98, P = 0.024). In contrast, social well-being (OR 0.96, 95% CI 0.83-1.11, P = 0.558) and social participation (OR 1.01, 95% CI 0.88-1.16, P = 0.849) were not significantly associated with SRH. Among the covariates, older age (OR 0.97, 95% CI 0.95-0.99, P = 0.019) and a greater number of physician-diagnosed conditions (OR 1.78, 95% CI 1.58-2.00, P < 0.001) were independently related to poorer perception, whereas sex was not significant (P = 0.085). 

## Discussion

This study aimed to identify the underlying components of health-related variables associated with SRH among community-dwelling older adults in Japan, using PCA and multivariable regression.

This study provides novel evidence of the multidimensional correlates of SRH among community-dwelling older adults in Japan. Using PCA and multivariable regression, we identified four distinct components: physical capability, social well-being, mobility patterns, and social participation. Together, these components explained approximately half of the variance across a wide range of health and lifestyle indicators. Among them, physical capability emerged as the most robust predictor of better SRH, whereas mobility patterns showed a modest negative association. By contrast, social well-being and social participation were not independently associated with SRH after adjusting for age, sex, and multimorbidity.

The primacy of physical capability aligns with prior studies demonstrating that mobility, strength, and functional independence are central to older adults’ health perceptions [23,24]. Physical function is readily observable in daily life and is strongly linked to autonomy and self-efficacy, which are psychological factors that foster positive health appraisals.

Contrary to expectations, social participation was not independently associated with SRH. This null finding may reflect mediation through physical function, which was already modeled [25], or cultural context. In Japan, where baseline community integration is relatively high [26], the incremental benefits of formal participation may be less pronounced than in societies with weaker community ties. It should also be noted that the present study did not include qualitative assessments of social relationships or community engagement. Although the survey captured participation frequency and contact levels, it did not explore the subjective quality or meaning of these interactions. Therefore, the limited association between social factors and SRH should be interpreted with caution, as the multidimensional nature of social well-being may not have been fully captured by the available indicators.

The negative association between mobility patterns and SRH suggests that reliance on public transportation may be perceived as a marker of declining independence [27], whereas maintaining the ability to use alternative transport modes, even mobility aids, may reinforce autonomy and thereby sustain positive health perceptions [28]. Future longitudinal studies are needed to clarify whether transportation reliance precedes poor SRH or vice versa.

Although social well-being loaded strongly on measures such as life satisfaction, trust, and perceived support, it did not independently predict SRH [29], once physical and medical factors were considered. This finding underscores the predominant role of functional health in shaping perceived health status in later life, even beyond psychosocial resources [30]. 

Several limitations should be noted. First, the cross-sectional design precludes any causal inference. Second, SRH was measured using a single-item indicator, which, although widely validated, may be influenced by cultural or individual response tendencies. Third, the study was conducted in a single municipality, which limits generalizability. Fourth, although the response rate (62.1%) was acceptable, the possibility of non-response bias cannot be ruled out; respondents may have been relatively healthier or more socially engaged than non-respondents, which could have influenced the observed associations. Finally, we did not include potentially relevant variables, such as cognitive function, healthcare access, or socioeconomic status. Nevertheless, these limitations do not diminish the robustness of the main finding that physical capability, as derived from PCA, remains the most salient determinant of SRH among older adults.

## Conclusions

In this large population-based study of older Japanese adults, physical capability emerged as the most important determinant of SRH, whereas reliance on public transportation was modestly associated with poorer perceived health. Social well-being and social participation, although conceptually important, did not demonstrate independent associations once physical function and multimorbidity were taken into account. These findings directly address the study’s primary objective of identifying the core components underlying SRH and clarifying their independent associations. They underscore the central role of physical function and mobility independence in shaping older adults’ perceptions of their health. Interventions that enhance motor fitness, support independent transportation, and address multimorbidity may represent pivotal strategies to improve SRH and promote well-being in aging populations. Given that SRH is a well-established predictor of morbidity and mortality, strengthening these domains may contribute to extending healthy life expectancy and guiding community-based health policy.

## Appendices

**Table 5:** List of variables included in the PCA The variables listed above represent the 58 questionnaire items analyzed in the PCA. These items comprehensively capture the physical, psychological, and social dimensions related to self-rated health among community-dwelling older adults. No statistical tests were performed for this table. PCA: principal component analysis

Variable
Feels bored every day
Prefers staying at home to going out or trying new activities
Feels that life is not worthwhile
Feels helpless
Satisfaction with life
Able to climb stairs
Able to climb stairs without shortness of breath
Able to jump
Able to run
Able to overtake others while walking
Able to continue walking for more than 30 minutes
Able to carry a bucket filled with water
Able to lift a 10-kg bag of rice
Able to lift a fallen bicycle
Able to open the lid of a wide-mouthed jar (e.g., jam jar)
Able to touch the floor from a standing position without bending the knees
Able to put on socks, trousers, or a skirt while standing without support
Able to stand up from a chair without using hands
Able to stand on tiptoe without holding onto anything
Able to go out alone using public transportation
Able to manage bank deposits and withdrawals
Able to provide advice or counsel to family members or friends
Able to shop for daily necessities
Usual mode of transportation when going out: Bicycle
Usual mode of transportation when going out: Car (as a passenger)
Usual mode of transportation when going out: Car (self-driving)
Usual mode of transportation when going out: Rollator (wheeled walker)
Usual mode of transportation when going out: Wheelchair
Usual mode of transportation when going out: Other
Usual mode of transportation when going out: Walking
Usual mode of transportation when going out: Motorcycle
Usual mode of transportation when going out: Bus
Usual mode of transportation when going out: Train
Usual mode of transportation when going out: Taxi
Usual mode of transportation when going out: Motorized four-wheeled vehicle (e.g., electric cart)
Number of relatives contacted at least once a month (by phone or in person)
Number of relatives with whom one can comfortably discuss personal matters
Number of relatives who can be relied upon for help in times of need
Number of friends contacted at least once a month (by phone or in person)
Number of friends with whom one can comfortably discuss personal matters
Number of friends who can be relied upon for help in times of need
People in the community can be trusted
People in the community are willing to help each other
Frequently interacts with people of different backgrounds in daily life
Frequency of participation in community events
Frequency of participation in neighborhood association activities
Frequency of participation in senior club activities
Frequency of participation in hobby groups
Frequency of activities involving sharing one’s skills or experiences with others
Frequency of participation in volunteer activities
Frequency of going outdoors
Frequency of exercise
Frequently interacts with people of similar background in daily life
Frequency of conversations with neighbors
Attachment to the community
Visits friends at their homes
Many neighbors are of a similar generation or lifestyle
Willingness to contribute to the local community
